# Impact of Uremic Toxins on Endothelial Dysfunction in Chronic Kidney Disease: A Systematic Review

**DOI:** 10.3390/ijms23010531

**Published:** 2022-01-04

**Authors:** Eva Harlacher, Julia Wollenhaupt, Constance C. F. M. J. Baaten, Heidi Noels

**Affiliations:** 1 Institute for Molecular Cardiovascular Research, University Hospital Aachen, Rheinisch-Westfälische Technische Hochschule Aachen University, 52074 Aachen, Germany; evstraussfel@ukaachen.de (E.H.); jwirth@ukaachen.de (J.W.); 2 Department of Biochemistry, Cardiovascular Research Institute Maastricht, Maastricht University, 6200 MD Maastricht, The Netherlands

**Keywords:** chronic kidney disease, uremic toxins, vascular dysfunction, vascular pathophysiology, endothelial cells, endothelial dysfunction, cardiovascular disease

## Abstract

Patients with chronic kidney disease (CKD) are at a highly increased risk of cardiovascular complications, with increased vascular inflammation, accelerated atherogenesis and enhanced thrombotic risk. Considering the central role of the endothelium in protecting from atherogenesis and thrombosis, as well as its cardioprotective role in regulating vasorelaxation, this study aimed to systematically integrate literature on CKD-associated endothelial dysfunction, including the underlying molecular mechanisms, into a comprehensive overview. Therefore, we conducted a systematic review of literature describing uremic serum or uremic toxin-induced vascular dysfunction with a special focus on the endothelium. This revealed 39 studies analyzing the effects of uremic serum or the uremic toxins indoxyl sulfate, cyanate, modified LDL, the advanced glycation end products N-carboxymethyl-lysine and N-carboxyethyl-lysine, p-cresol and p-cresyl sulfate, phosphate, uric acid and asymmetric dimethylarginine. Most studies described an increase in inflammation, oxidative stress, leukocyte migration and adhesion, cell death and a thrombotic phenotype upon uremic conditions or uremic toxin treatment of endothelial cells. Cellular signaling pathways that were frequently activated included the ROS, MAPK/NF-κB, the Aryl-Hydrocarbon-Receptor and RAGE pathways. Overall, this review provides detailed insights into pathophysiological and molecular mechanisms underlying endothelial dysfunction in CKD. Targeting these pathways may provide new therapeutic strategies reducing increased the cardiovascular risk in CKD.

## 1. Introduction

As kidney function gradually declines, the risk of cardiovascular complications increases. This is reflected by the fact that approximately half of the patients with severe chronic kidney disease (CKD stage 4–5) die from cardiovascular disease (CVD) [1], compared to 26% of patients with a healthy kidney function [2,3]. Aortic valve stenosis, left ventricular hypertrophy, myocardial ischemia and heart failure are the leading causes of death in CKD patients [4]. As main underlying mechanism of myocardial ischemia, CKD patients are at increased risk for atherosclerosis, an inflammatory process within the intimal layer of the vessel wall [5]. In CKD, the formation and progression of such atherosclerotic lesions is highly accelerated [6]. Moreover, as CKD progresses, vascular stiffness increases [7], with vascular stiffness as an important predictor of cardiovascular mortality in CKD patients [8]. 

Endothelial dysfunction underlies both atherosclerosis and vascular stiffness and is associated with an increased risk of cardiovascular death [9]. Coronary endothelial-dependent vasoreactivity was identified as a predictor of future cardiovascular events and disease progression [10]. 

**Figure 1:**
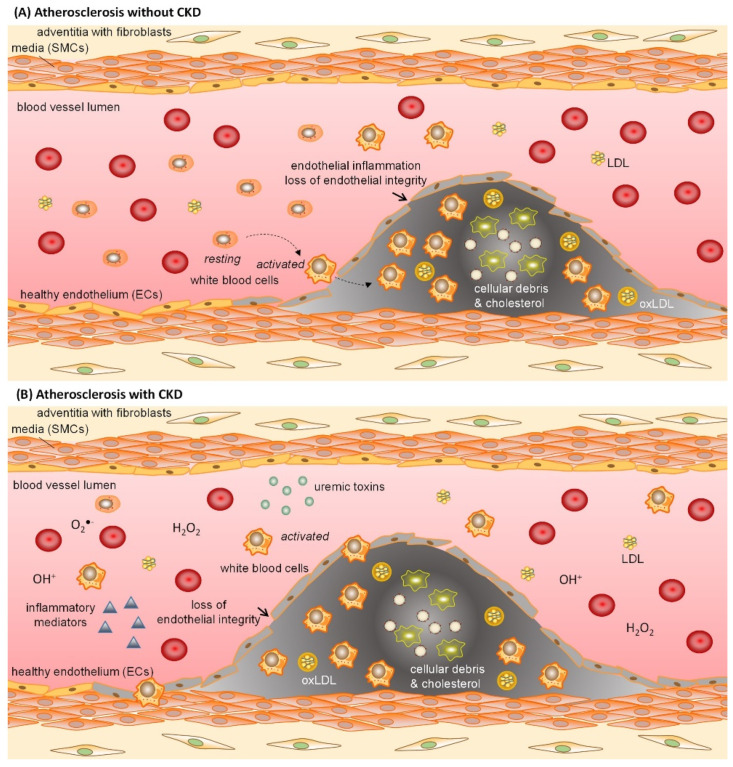
Endothelial inflammation and loss of endothelial integrity contribute to endothelial dysfunction and the formation of atherosclerotic lesions in the vessel wall. Atherosclerosis is characterized by endothelial inflammation and an increased vascular permeability. Inflamed endothelial cells secrete chemokines that recruit white blood cells, which migrate through the dysfunctional endothelial barrier into the vessel wall. Moreover, LDL migrates into the vessel wall where it is oxidized to oxLDL (**A**). In patients with CKD, the process of atherosclerotic lesion formation is advanced because of CKD-associated factors such as systemic chronic low-grade inflammation, increased markers of oxidative stress and lipoprotein oxidation, as well as the accumulation of uremic toxins, all promoting damage to the vessel wall (**B**). ECs = endothelial cells; LDL = low density lipoprotein; oxLDL = oxidized LDL; SMCs = smooth muscle cells.

Atherosclerotic lesions start out as patches of dysfunctional endothelial cells (ECs) [11]. As the endothelial barrier becomes dysfunctional and loses its integrity, permeability increases and low-density lipoproteins (LDL) accumulate within the vessel wall, upon which LDL becomes oxidized and an inflammation process is initiated [12]. Chemokines are secreted and adhesion molecules are expressed by inflamed ECs, triggering leukocyte recruitment, their adhesion on ECs as well as their migration into the vessel wall [11,13]. Furthermore, increased oxidative stress and reactive oxygen species (ROS) production reduce the bioavailability of nitric oxide (NO) [13], which in turn causes a reduction in EC-dependent vasodilation and as such an increase in vascular stiffness. Concomitantly, dysfunctional ECs lose their antithrombotic properties and prothrombotic properties prevail, further increasing the risk of atherothrombosis [14]. Moreover, dysfunctional ECs show reduced survival and proliferation capacity, processes needed to restore the protective endothelial barrier and counteract injury-induced stenosis after vascular injury [15,16]. Combined, pathophysiological processes of inflammation, oxidative stress as well as impaired survival, proliferation and repair of ECs contribute to endothelial dysfunction and CVD (Figure 1A).

Several studies indicate that CKD-associated factors, such as systemic chronic low-grade inflammation, increased oxidative stress and uremic toxins accelerate atherosclerosis in CKD (Figure 1B), although the underlying molecular mechanisms are not fully understood [17,18]. CKD patients exhibit a systemic, chronic low-grade inflammation as well as increased oxidative stress even in early stages of CKD [18,19,20], characterized by high levels of circulating inflammatory proteins (CRP, IL6) and oxidative stress biomarkers [19]. The more CKD progresses, the more oxidative stress levels increase [20]. Further, it was shown that markers of oxidative stress (including lipid peroxidation markers and lipoprotein oxidation propensity, among others) inversely correlated with endothelial-dependent vasodilation in CKD patients, independent of classical risk factors of atherosclerosis such as gender, age, blood pressure, diabetes and lipid-lowering treatment [21]. Modification of lipids, such as oxidation of LDL, is increased in CKD and further promotes vascular damage [22]. Furthermore, high phosphate levels typical of CKD-related mineral bone disorders cause endothelial dysfunction by changing EC morphology, decreasing viability, and promoting senescence [6]. 

Upon kidney dysfunction, solutes accumulate in the circulation that are normally excreted by the kidneys. The accumulation of these uremic toxins causes a gradual endogenous intoxication. In literature, more than 140 uremic toxins have been described to be elevated upon kidney dysfunction [23,24]. Uremic toxins such as indoxyl sulfate (IS) and p-cresyl sulfate (pCS) have been associated with an increased risk of cardiovascular events and cardiovascular mortality in CKD patients. Mechanistically, many of these uremic toxins have been linked to inflammation and oxidative stress [25,26], but also to arterial stiffness and endothelial dysfunction in vitro [27]. Given the central role of the endothelium in preserving vascular health and counteracting atherosclerosis and cardiovascular risk, this manuscript systematically reviewed uremic toxin-induced EC dysfunction and its implications for cardiovascular disease specifically in the context of CKD.

## 2. Materials and Methods

This systematic review is in accordance with the guidelines provided by the PRISMA statement [28]. The completed PRISMA checklist can be found in the Supplemental Materials (Table S2). 

### 2.1. Search Strategy

An advanced literature search was performed in PubMed and Web of Science for studies describing the mechanisms underlying vascular pathophysiology in CKD, with a special focus on CKD-induced endothelial dysfunction. Additionally, to compare pathological signaling processes within different cell types present in atherosclerotic plaques, studies on CKD-induced dysfunction of smooth muscle cells, monocytes and macrophages were retrieved. Studies published until June 2021 were assessed. Supplemental Table S2 provides an overview of the terms and conditions that were used in the literature search.

### 2.2. Study Selection Criteria

**Figure 2:**
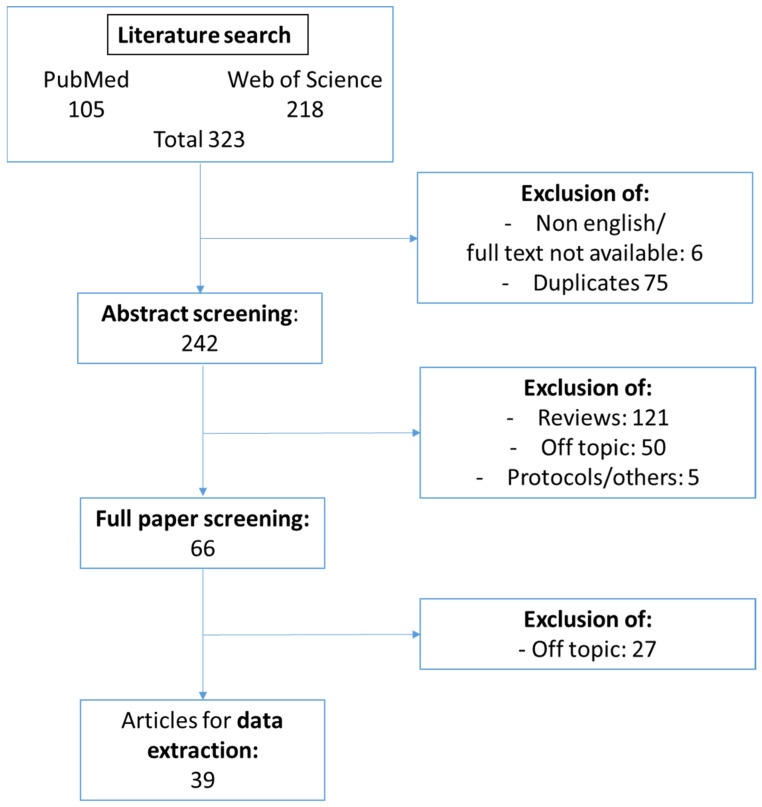
Flow diagram of study selection. Flow diagram of the selection of original papers illustrating the number of in- and excluded studies throughout the selection process.

Two reviewers (E.H. and J.W.) independently selected studies presenting original data using predefined eligibility criteria. Studies that described mechanisms responsible for CKD-induced vascular dysfunction were included for further analysis. These studies either described the effect of uremic serum or individual uremic toxins on endothelial function. Reports on the effects of CKD on different cell types present within the atherosclerotic plaque were also taken along. In case markers of endothelial function or uremic toxin-induced signaling pathways were measured in CKD patient populations, these patient studies were incorporated as well. Duplicates, review papers, poster abstracts and papers not written in the English language were excluded as well as studies that concentrated on CKD-induced vascular calcification or studies in which mechanistic insight was lacking. Both reviewers had to agree on inclusion. In case of disagreement, a third reviewer (C.B.) was consulted to achieve consensus. A graphical overview of the number of in- and excluded studies throughout the selection process is presented in Figure 2.

### 2.3. Data Extraction

To summarize the pathophysiological effects of uremia or individual uremic toxins on endothelial cells, studies were classified based on the toxins investigated and their key effects on endothelial function. These key effects were defined as inflammation, oxidative stress, cell death, leukocyte adhesion and migration, cell proliferation and thrombosis. For a description of molecular mechanisms underlying uremic toxin-induced endothelial dysfunction, information on signaling pathways was extracted from in vivo and in vitro studies. From patient studies, information on markers for endothelial function or insights into uremic toxin-induced signaling pathways was extracted. 

## 3. Results and Discussion

### 3.1. Study Selection

Using predefined literature search terms (Table S1), our search initially included 323 articles (105 publications identified via PubMed and 218 from Web of Science). After excluding non-English publications and duplicates, 242 publications were left. Through abstract screening, 176 publications were excluded as being either reviews or out of scope. Of the remaining 66 papers, the full text was screened. In total, 39 publications were included in the systematic review and mechanistic data were extracted. The selection procedure is shown in Figure 2.

### 3.2. Pathophysiological Effect of Uremic Toxins on Endothelial Cells

To provide insights into how a reduced kidney function may impact endothelial function, our literature search retrieved studies in which endothelial cells were treated with uremic serum or with individual uremic toxins. 

Uremic serum triggered processes of oxidative stress, cell adhesion as well as inflammation. Decreased antioxidant enzyme activity as well as elevated lipid peroxidation indicated redox imbalances in endothelial cells treated with uremic serum [29]. Further, in endothelial cells, oxidative stress was induced by uremic serum as reflected by an increase in the formation of reactive oxygen species (ROS) [30,31] via a RAGE-NF-κB dependent pathway [30]. RAGE-NF-κB signaling as well as glutathione S-transferase μ1 (GSTM-1), which is a downstream gene in the Aryl-Hydrocarbon-Receptor (AhR) signaling pathway, were also responsible for a uremic serum-mediated increase in endothelial expression of adhesion molecules [29,30]. Among inflammatory mediators dysregulated in CKD [29], increased levels of VCAM-1 and the pro-inflammatory cytokine MCP-1 in uremic endothelial cells led to increased monocyte adhesion and thus increased inflammation [30,32]. 

Among the uremic toxins studied for direct effects on endothelial cells, our literature search identified studies investigating indoxyl sulfate, cyanate, the advanced glycation end products (AGEs) N-carboxymethyl-lysine (CML) and N-carboxyethyl-lysine (CEL), p-cresol and p-cresyl sulfate, uric acid and asymmetric dimethylarginine (ADMA) [33,34]. Furthermore, phosphate and post-translationally modified LDL, both with increased levels in advanced CKD [22,35,36], were studied for direct effects on endothelial cells. Table 1 summarizes the effect of these molecules on endothelial cells, classified in effects on inflammation, oxidative stress, leukocyte adhesion and migration, proliferation, cell death and thrombosis. 

### 3.3. Identification of Molecular Mechanisms Underlying Uremic Toxin-Induced Endothelial Dysfunction

#### 3.3.1. Tryptophan-Derived Toxins Trigger Inflammation and Oxidative Stress in Endothelial Cells

**Figure 3:**
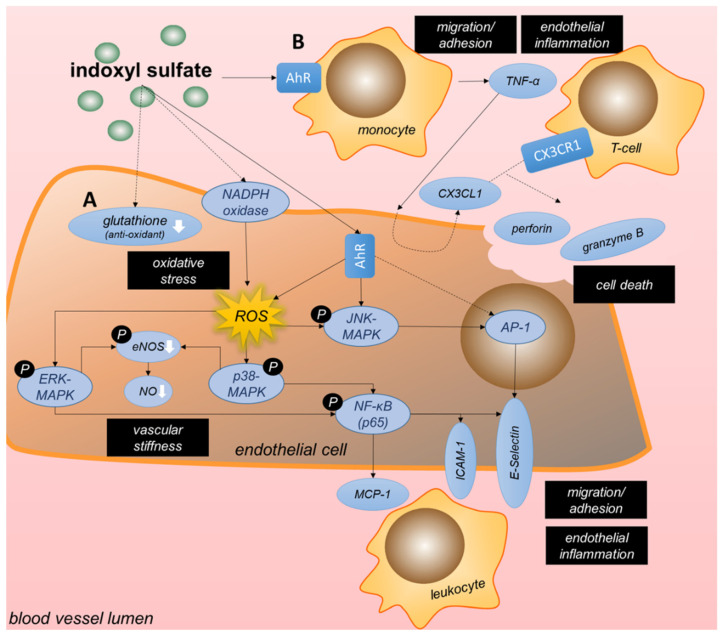
Mechanisms underlying indoxyl sulfate-induced endothelial dysfunction. The uremic toxin indoxyl sulfate (IS), which highly accumulates in CKD patients, leads to endothelial dysfunction by enhancing oxidative stress, endothelial inflammation, recruitment and adhesion of inflammatory cells, cell death as well as by reducing endothelial proliferation. In addition, it reduces endothelial nitric oxide production, contributing to enhanced vascular stiffness. As underlying mechanisms, IS induces the production of reactive oxygen species (ROS) and binds to the Aryl Hydrocarbon Receptor (AhR), thereby enhancing intracellular signaling through MAP Kinases (ERK, p38, JNK) and NF-κB (p65), triggering pro-inflammatory and apoptotic responses in endothelial cells (**A**). In addition, IS-AhR pro-inflammatory activation of monocytes contributes to endothelial dysfunction (**B**). With the focus on the cell signaling pathways, the figure does not take into account a differentiation between basal and apical side of the cell. AhR = Aryl Hydrocarbon Receptor, AP-1 = activator protein-1; eNOS = endothelial nitric oxide synthase; MAPK = mitogen activated protein kinase; NF-κB = nuclear factor kappa B; NO = nitric oxide; p-eNOS = phosphorylated, active eNOS; ROS = reactive oxygen species; TNF = tumor necrosis factor.

Tryptophan-derived indoxyl sulfate (IS), a protein-bound uremic toxin which highly accumulates in CKD patients, and which can only to a minor extent amount be removed through dialysis, has been frequently investigated in the context of CKD-induced endothelial dysfunction. Of the 11 papers identified through our literature search that report on IS-induced signaling and cellular effects, 8 describe the effects of IS on endothelial function. Three studies reported on different cell types, one of which examined the crosstalk between the effects of IS on macrophages and endothelial dysfunction. Oxidative stress, inflammation, cell death as well as reduced proliferation are central to IS-induced endothelial dysfunction (Table 1) [31,37,38,39,40,41,42,43]. The main mechanisms and the contributing signaling pathways are summarized in Figure 3.

Six out of eight studies detected an increase in ROS levels and thus oxidative stress upon IS treatment of endothelial cells [31,37,38,39,41,42]. Here, oxidative stress was induced by activating NADPH oxidase [38,41], reducing levels of the antioxidant glutathione [41] or by triggering the AhR pathway [37] (Figure 3A). Subsequently, these high levels of IS-induced ROS resulted in an upregulated expression of MCP-1 and ICAM-1, prominent mediators of inflammatory cell recruitment and adhesion, by ROS-dependent ERK/MAPK-induced activation of NF-κB signaling [38,39,42]. But not only is IS linked to inflammation in CKD patients, tryptophan-derived 3-hydroxyanthranilic acid has also been associated with levels of the pro-inflammatory chemokines MCP-1 as well as macrophage inflammatory protein-1β (CCL4) in CKD patients [62]. Contributing to IS-induced oxidative stress, IS also decreased NO production and eNOS phosphorylation in endothelial cells via the ROS-ERK/MAPK pathway [42], although without effects on mRNA expression of eNOS [41]. Additionally, high ROS levels upon IS were shown to enhance TNF-α-induced E-selectin expression by increasing NF-κB activation and AP-1 over JNK phosphorylation [37]. The antioxidants vitamin C, N-acetylcysteine and vitamin E could reduce IS-induced oxidative stress by preventing the activation of NADPH in a dose-dependent fashion [41]. Klotho protein was identified as another negative regulator of IS-induced ROS formation, and could also abrogate the reduction in eNOS phosphorylation and NO production observed in endothelial cells upon IS-induced p38/MAPK-NF-κB signaling [42]. Furthermore, the kinases integrin linked kinase (ILK) and AKT, whose activity was upregulated by IS, counteracted IS-induced ROS production and apoptosis of endothelial cells as negative feedback mechanism [31], although the underlying mechanisms were not investigated.

As introduced above, IS exposure also activates the AhR pathway. AhR is a ligand-activated transcription factor expressed in macrophages, monocytes [40,63] and endothelial cells [64]—amongst other cells—and its activation can trigger an inflammatory response. For example, stimulation with AhR agonists has been shown to increase cholesterol levels in macrophages and to trigger the release of various inflammatory markers such as IL-8 and MCP-1 [65,66]. Further, activation of the AhR pathway worsens atherosclerosis in mice [67]. Amongst other ligands, AhR signaling is triggered by IS, as shown in endothelial cells [37], monocytes [40] and macrophages [68,69] in vitro. As underlying mechanism of pro-atherogenic effects of AhR signaling in endothelial cells, IS increased leukocyte-endothelium interactions in TNF-α-treated mice through endothelial AhR signaling. In vitro, IS-AhR signaling upregulated endothelial E-selectin expression through activator protein-1 (AP-1) transcriptional activity [37]. Of note, other tryptophan-derived uremic toxins have also been shown to interact with AhR, such as indole acetic acid (IAA), which triggers endothelial inflammation and oxidative stress via AhR-dependent signaling [70]. AhR expression on peripheral blood mononuclear cells (PBMCs) from CKD patients positively correlated with plasma levels of IAA [71].

Besides its inflammatory effects, increased IS levels have been shown to cause apoptosis of endothelial cells via the COX-2/PGE_2_ axis, which was upregulated by a decreased expression of miR-214. Subsequently, PGE_2_ increased the expression of caspase-3 and BAX and caused an increase in the levels of cleaved caspase-3 in mouse aortic endothelial cells [43]. However, studies in human umbilical vein endothelial cells contradicted this effect on caspase-3 protein expression and apoptosis in response to similar amounts of IS [42]. Another study revealed an indirect effect of IS on endothelial apoptosis through pro-inflammatory responses in monocytes and subsequent T-cells. Moreover, in monocytes and macrophages, IS can activate the AhR pathway, which mediated the secretion of high concentrations of TNF-α [40,69] via crosstalk between the NF-κB and AhR pathways. These increased levels of TNF-α subsequently initiated CX3CL1 production in endothelial cells [40]. This triggered the recruitment and activation of CX3CR1-expressing T-cells with subsequent secretion of cytotoxic granules containing perforin and granzyme B, ultimately causing apoptosis of endothelial cells [40] (Figure 3B). In patients with kidney failure, increased plasma CX3CL1 levels have been detected [40] with higher CX3CL1 levels associated with an increased overall mortality as well as a higher prevalence of CVD in a CKD cohort [72]. In macrophages, IS also increased pro-IL-1β mRNA expression through NF-κB/p65 signaling, however, this was without effect on mature IL-1β protein levels due to IS-mediated reduction in NLRP3 expression [68]. Finally, in contrast to the pro-apoptotic effect of IS on endothelial cells discussed above, it was reported that vascular smooth muscle cells (SMCs) stimulated with IS were more proliferative, with the pro-proliferative cyclin D1 and p21, and the anti-apoptotic p53 upregulated in a glucose transporter-1 (GLUT1) dependent fashion. IS reduced the phosphorylation of AKT and TSC2, thereby increasing mTOR/S6K signaling to drive GLUT1 expression [73].

#### 3.3.2. Cyanate Triggers Inflammatory Signaling and Protein Carbamylation

**Figure 4:**
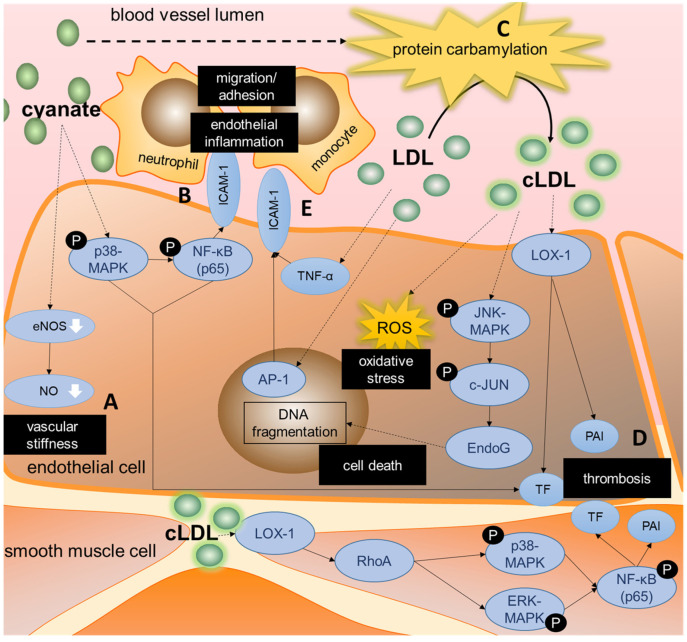
Mechanisms underlying cyanate or (modified) LDL-induced endothelial dysfunction. Increased cyanate levels as in CKD patients induce cellular signaling triggering endothelial dysfunction as displayed in increased vascular stiffness (**A**) as well as enhanced endothelial inflammation, triggering the recruitment and adhesion of inflammatory cells (**B**). Furthermore, cyanate-induced protein carbamylation leads to carbamylated low-density lipoprotein (cLDL), which can trigger oxidative stress and cell death (**C**) and can increase thrombotic risk through increases in tissue factor and PAI (**D**). In addition, LDL was shown to contribute to endothelial inflammation by upregulating pro-inflammatory cytokines and adhesion molecules, triggering inflammatory cell recruitment and adhesion (**E**). AP-1 = activator protein-1; cLDL = carbamylated LDL; EndoG = endonuclease G; eNOS = endothelial nitric oxide synthase; ICAM = intracellular adhesion molecule; LDL = low density lipoprotein; LOX-1 = Lectin-like oxidized low density lipoprotein receptor-1; MAPK = mitogen activated protein kinase; NF-kB = nuclear factor kappa B; NO = nitric oxide; PAI = plasminogen activator inhibitor; TF = tissue factor; TNF = tumor necrosis factor.

In CKD patients, cyanate levels can rise to three times the normal levels resulting from the conversion of the uremic toxin urea, which levels are also highly increased in CKD [33,74,75]. Another endogenous source of cyanate is the oxidation of thiocyanate by the phagocyte protein myeloperoxidase (MPO) [76,77]. High local MPO concentrations have been found in the subendothelial matrix of inflamed vascular tissue, which suggests that vascular endothelial cells are particularly exposed to high cyanate levels [44,78]. Six publications identified by our literature search examined the relevance of cyanate in context of CKD-induced endothelial dysfunction (Table 1). The mechanisms are summarized in Figure 4.

The important role of cyanate in the development of endothelial dysfunction and vascular inflammation in CKD patients was shown in two publications by El Gamal et al. [44,45]. They showed that cyanate-treated mice produced lower nitrite levels and displayed decreased acetylcholine-induced vasorelaxation [45]. Mechanistically, cyanate reduced eNOS protein levels and its phosphorylation in mouse aortas, contributing to a decreased NO production [45] (Figure 4A). Further, in vitro as well as in vivo, cyanate induced the expression of ICAM-1 in endothelial cells by activating the p38-MAPK and NF-κB pathways. This triggered enhanced neutrophil adhesion, thereby contributing to vascular inflammation [44] (Figure 4B). In patients with kidney failure, ICAM-1 plasma levels correlated with the carbamyllysine level as marker of cyanate formation [44]. 

Furthermore, urea-derived cyanate is known to mediate post-translational ‘carbamylation’ of proteins [79], as for example demonstrated for low-density lipoproteins (LDL) [44]. Carbamylated LDL (cLDL) is associated with endothelial injury and atherosclerosis in mice [35,80,81,82,83,84,85]. Aposotlov et al. showed that in vitro, elevated cLDL-levels led to endothelial mitotic cell death by DNA fragmentation [48]. They described caspase-independent signaling via JNK/c-JUN, resulting in DNA fragmentation by endonuclease G [48] (Figure 4C). Their data suggested that other DNA-destroying mechanisms are also involved, with a role of Mitogen-activated protein kinase (MEK) and ERK1/2 kinases, but precise details are unknown at present [48].

In regard to atherothrombosis, in vitro cyanate was shown to upregulate the expression of tissue factor and plasminogen activator inhibitor-1 (PAI-1) in endothelial cells, key mediators in mediating coagulation and inhibiting fibrinolysis [45] (Figure 4D). Furthermore, both mRNA expression and activity of tissue factor and PAI-1 were upregulated in arterial tissue and plasma of mice treated with cLDL as well as in vitro in aortic SMCs and endothelial cells [47]. In aortic SMCs, the increase in tissue factor and PAI-1 was mediated by binding of cLDL to the oxidated LDL-receptor LOX-1 and the subsequent activation of the RhoA/ERK/p38/NF-κB pathway (Figure 4D). In whole blood of cLDL-treated mice, the upregulation of tissue factor led to increased thrombin generation. In addition to effects of cLDL on the coagulation cascade, cLDL also enhanced agonist-induced platelet aggregation by phosphorylating p38 and translocating LOX-1 to the platelet surface [47].

As well as this, unmodified LDL was shown to cause endothelial inflammation by, among other processes, increasing monocyte adhesion [46]. LDL increased the nuclear binding activity of AP-1 in endothelial cells, which in turn upregulated TNF-α and ICAM-1 expression mediating monocyte adhesion [46] (Figure 4E). LDL can be divided into two subclasses based on size, CKD patients have increased levels of small dense LDL compared to healthy controls [22,86]. Ambrosch et al. differentiated between these LDL subtypes and showed that the smaller LDL particles (diameter < 25.5 nm) caused a stronger increase in AP-1 binding activity in HUVECs and subsequent TNF-α and ICAM1 expression compared to the larger LDL particles (diameter > 25.5 nm), resulting in more monocyte adhesion [46].

Besides carbamylation, other post-translational modifications of lipoproteins have also been studied in the context of CKD and CVD [12,22]. Our search revealed a study by Tao et al., who investigated the mechanisms behind acetylated LDL (AcLDL)-induced apoptosis in macrophages [87]. Endoplasmic reticulum stress with increased CHOP and decreased B-cell lymphoma-2 (BCL-2) expression was suggested to be a key mediator of AcLDL-induced macrophage apoptosis [87]. 

#### 3.3.3. AGEs Are an Important Group of Uremic Toxins Inducing Endothelial Dysfunction

**Figure 5:**
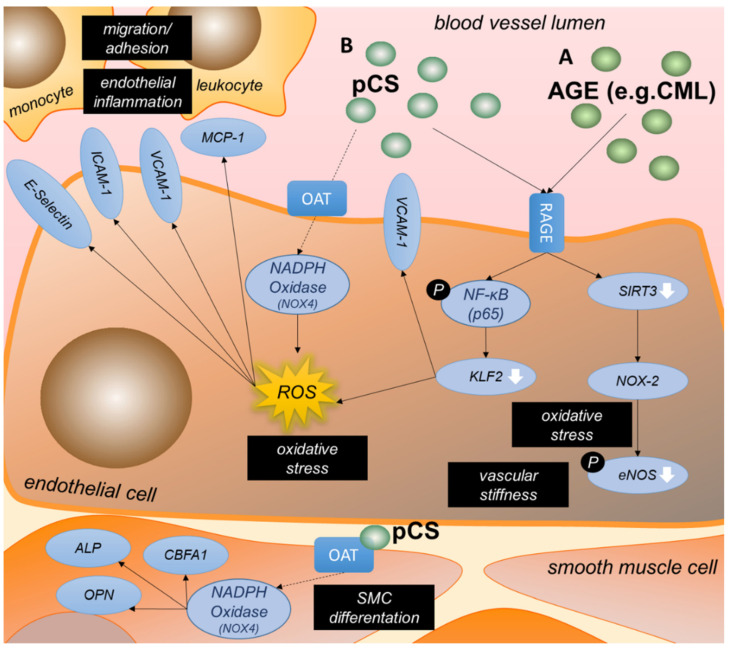
Mechanisms underlying p-cresyl sulfate or AGE-induced endothelial dysfunction. By activating RAGE, AGEs mediate oxidative stress and reduce vasoreactivity by decreasing eNOS activation in endothelial cells. Further, AGEs as well as p-cresyl sulfate increase ROS generation via KLF2. ROS increase endothelial inflammation by enhancing surface expression of chemokines (e.g., MCP-1) and surface adhesion proteins (e.g., VCAM-1, ICAM-1, E-Selectin), triggering leukocyte recruitment and binding (**A**). Cellular uptake of p-cresyl sulfate by endothelial cells as well as SMCs takes place via organic anion transporters. This results in the activation of NADPH oxidase NOX-4, leading to the differentiation of SMCs and ROS generation in endothelial cells (**B**). ALP = alkaline phosphatase; CBFA = core-binding factor alpha; eNOS = endothelial nitric oxide synthase; ICAM = intracellular adhesion molecule; KLF = krüppel-like factor; MCP = monocyte chemoattractant protein; NADPH = nicotinamide adenine dinucleotide phosphate; NF-κB = nuclear factor kappa B; OAT = organic anion transporters; OPN = osteopontin; ROS = reactive oxygen species; SIRT = sirtuin; NOX = NADPH oxidase; SMC = smooth muscle cell; VCAM = vascular cell adhesion molecule.

Advanced glycation end (AGE) products belong to the protein-bound uremic toxins and are highly increased in CKD [88]. AGEs are proteins or protein degradation products that become post-translationally modified upon exposure to sugars. The transmembrane receptor for advanced glycation end products (RAGE) is a cell surface marker involved in cell migration, adhesion, and oxidative stress [30,49,50,51]. Ten papers from our search discussed the AGE—RAGE signaling pathway in the context of uremic toxin-induced endothelial dysfunction. RAGE as well as its binding partners were shown to have a pro-inflammatory and pro-oxidative effect in CKD and as such to promote endothelial dysfunction. Mechanisms are summarized in Figure 5A.

It was demonstrated by three groups that AGEs (including protein bound N-carboxymethyl-lysine (CML) and methyl-glyoxal (MG)) induced RAGE-signaling and enhanced RAGE expression in endothelial cells [30,49,50]. In CKD patients stage 1–5, glomerular filtration rate as measure of kidney function was inversely correlated with RAGE mRNA levels in PBMCs as well as total CML levels in serum [50]. RAGE-mediated ROS formation is reported in response to AGEs in endothelial cells [30]. CML-mediated RAGE signaling in endothelial cells decreased endothelial expression of transcription factor Krüppel-like factor 2 (KLF2) via NF-κB, and subsequently increased ROS production. These effects of CML were enhanced with exposure of endothelial cells to shear stress [30]. Further, it was shown that AGEs inhibit eNOS expression [50] as well as eNOS phosphorylation via RAGE in endothelial cells [49]. As underlying mechanism, RAGE activation by AGEs decreased SIRT3 expression, which in turn led to mitochondrial oxidative stress. Consequently, NOX-2 activity was upregulated, increasing cytosolic oxidative stress and thereby reducing eNOS phosphorylation [49]. The reduction in KLF2 expression also resulted in an increased VCAM1 surface expression and as such supported monocyte adhesion [30]. However, in hemodialysis patients, plasma levels of the AGEs CML and MG did not correlate with increased circulating levels of VCAM-1 [89] and neither with soluble ICAM levels [44].

On top of this, non-RAGE related cellular effects of AGEs toxins, such as CML and N-carboxyethyl-lysine (CEL), were identified in our literature search. Zhu et al. identified CML and CEL to induce endothelial inflammation and oxidative stress independently of RAGE signaling, and also revealed these AGEs to negatively influence endothelial progenitor cell (EPC) adhesion and proliferation [51], thus further contributing to a dysfunctional endothelium.

#### 3.3.4. P-Cresyl Sulfate Contributes to Oxidative Stress and Inflammation in Endothelial Cells

P-cresol occurs mainly in its sulfate conjugated form (>95%), called p-cresyl sulfate (pCS) [90]. It is this protein-bound uremic toxin that is identified to be associated with CVD in hemodialysis patients [91]. Five papers from our literature search discussed the effects of p-cresol or pCS on endothelial function. 

Three studies showed a connection between pCS, oxidative stress and endothelial dysfunction (Figure 5B). Not only AGEs, as discussed above, but also pCS binds to RAGE, thereby activating NF-κB signaling in endothelial cells and as such decreasing KLF2 expression [30]. pCS stimulation of endothelial cells also resulted in NADPH oxidase-mediated ROS formation, independent of RAGE, and mainly mediated by free pCS rather than protein-bound pCS. Especially in human aortic SMCs the effect appeared to be induced by free pCS, as the addition of albumin reduced the effect by 90% [53]. pCS accumulates in the kidney via the organic anion transporters (OAT) receptor [92]. pCS was also shown to be absorbed by vascular endothelial and smooth muscle cells via OAT and subsequently enhanced expression of NOX-4, a component of NADPH oxidase. This triggered endothelial MCP-1 expression in vascular SMCs, as well as upregulated the expression of alkaline phosphatase (ALP) as early phenotypic marker of osteoblast differentiation [53]. Furthermore, an increase in intracellular pCS via OAT is directly associated to osteogenic SMC differentiation, as shown by high expression of other osteoblast-specific proteins osteopontin (OPN) and core-binding factor alpha 1 (CBFA1) as well as activation of ALP. In pCS-treated 5/6-nephrectomized rats this connection of MCP-1 levels in serum, vascular OPN expression, and vascular ALP activity accompanied by the induction of NOX-4 expression could be confirmed [53]. Moreover, Jing et al. confirmed the pCS-induced increase of NOX-4 via ROS in endothelial and smooth muscle cells in vivo and in vitro and in addition showed an increase in NOX-1 and p22phox, proteins involved in ROS production [52]. 

pCS also affected leukocyte adhesion and migration. Increases in NOX-1, NOX-4 and p22phox by pCS led to higher levels of MCP-1, TNF-α, ICAM, VCAM as well as E-Selectin in endothelial cells in vitro, with increased expression of these pro-inflammatory chemokines and adhesion molecules also confirmed in aortic tissue of ApoE^−/−^ mice with CKD and pCS treatment [52]. In hemodialysis patients, pCS was shown to be an independent risk factor for the presence of carotid atherosclerotic plaque [52]. Furthermore, in hemodialysis patients, the increase in free and total p-cresol was reported to correlate with the number of endothelial microparticles (EMPs) and shed EMPs. In vitro, the Rho kinase pathway was shown to be mediating this increase in shed EMPs and EMP numbers in response to both p-cresol and pCS as well as uremic serum [54].

Finally, Garcia-Jerez et al. showed that in addition to uremic serum and IS, also p-cresol induced ROS-mediated apoptosis of endothelial cells. However, similarly as shown for IS, p-cresol also triggered a protective feedback mechanism by activating integrin-linked kinase (ILK) via an unknown pathway [31], thereby upregulating AKT phosphorylation at Ser^471^ and as such counteracting p-cresol-induced ROS and apoptosis [31].

#### 3.3.5. Phosphate Reduces NO Production and Triggers Inflammation in Vascular Cells

In CKD patients serum phosphate levels are increased and associated with increased cardiovascular risk and mortality [93,94]. Hyperphosphatemia (>3 mM) is associated with inflammation and oxidative stress and contributes to endothelial dysfunction as well as to calcium deposition in SMCs [55,57,95,96]. 

The effect of high phosphate levels in the context of CKD and CVD was addressed in four publications within our systematic literature search [55,56,57,96]. In addition to hyperphosphatemia, one of these papers also examined the endothelial effects of low phosphate in comparison.

Phosphate levels that deviated upwards or downwards from the physiological value of 1 mM led to reduced NO production in endothelial cells [55]. Exposure of endothelial cells to increased extracellular phosphate resulted in increased intracellular phosphate concentrations mediated by sodium-dependent Pi transporters (PiT1/PiT2) [57]. Hyperphosphatemia reduced intracellular calcium and increased PKCβ2 signaling, leading to decreased eNOS abundance and reduced NO production in endothelial cells [55]. As well as this, hypophosphatemia reduced NO production [55]. On signaling level, hyperphosphatemia triggered a downregulation of PI3K/AKT/NF-κB and MAPK/NF-κB pathways in endothelial cells, whereas in hypophosphatemia these pathways were increased [55].

Additionally, hyperphosphatemia as well as hypophosphatemia negatively affected cell viability of endothelial cells [55]. High phosphate levels were associated with a decreased BCL-XL/BAX ratio, and although low phosphate levels increased this ratio, also hypophosphatemia increased the number of apoptotic endothelial cells [55]. Hsu et al. described an inhibition of AKT/mTOR phosphorylation caused by increased phosphate in endothelial cells, with a subsequent increase in autophagic activity [56]. Increased autophagic activity was confirmed in CKD rats along with increased cleaved caspase 3 as apoptotic marker in endothelial cells [56], with inhibition of autophagy further increasing the amount of apoptotic cells [56]. 

Moreover, in endothelial cells, elevated phosphate levels increased membrane blebbing and enhanced the release of procoagulant endothelial microparticles. Microparticles from phosphate-treated cells were comparable in terms of total protein amount when compared to microparticles released from healthy cells, but given the increase in phosphatidyl serine exposure, phosphate-induced microparticles were more supportive of thrombin generation [57]. Apoptosis and oxidative stress were excluded as the major cause of microparticle release. Protein phosphorylation differed in phosphate-treated ECs, based on which metabolic stress was suggested as a potential cause of membrane blebbing. Abbasian et al. hypothesized that hyperphosphatemia led to hyperphosphorylation of phosphatases such as PTPase and PSPase, decreasing their activity indicated by a decreased phosphorylation of Tropomyosin-3 (TM-3) [57]. 

Finally, in vascular SMCs, hyperphosphatemia led to an increased ROS formation, which in turn led to enhanced formation of inflammatory proteins including TNF-α, ICAM-1, IL-6, IL-1ß and IL-8, contributing to increased vascular inflammation [96]. 

#### 3.3.6. High ADMA Levels as in CKD Lead to Increased Endothelial Cell Death

High levels of asymmetric dimethylarginine (ADMA) have been associated with an increased cardiovascular risk [58,97,98]. Our systematic literature search revealed one study that examined ADMA in the context of endothelial dysfunction (Table 1), showing that in vitro treatment of endothelial cells with ADMA-induced apoptosis [58]. Endoplasmic reticulum (ER) stress increased as cytosolic Ca^2+^ levels rose and ER Ca^2+^ levels declined due to the inhibition of the sarco/endoplasmic reticulum calcium-ATPase [58]. Further, increased phosphorylation of protein kinase RNA-like ER kinase (PERK) and inositol requiring enzyme-1 (IRE1) was observed, both known to be associated with cell death [99,100]. Additionally, ADMA treatment of endothelial cells enhanced levels of the ER chaperone 78-kDa glucose-regulated protein (GRP78/BiP), indicating defective protein folding and degradation. Together with decreased levels of apoptosis inhibitor BCL-2 and increased cleavage of caspase-3, a critical executioner of apoptosis, ADMA was shown to be an inducer of apoptosis in endothelial cells [58].

#### 3.3.7. Uric Acid Increases Oxidative and ER Stress as well as Endothelial Cell Apoptosis

Uric acid, a water-soluble uremic toxin that is the end product of purine metabolism, was discussed in three publications identified in our systematic literature search. Uric acid levels are highly increased in patients suffering from CKD stage 4–5 even after hemodialysis [101]. Hyperuricemia contributes to the genesis and progression of CVD and CKD [102,103], with as underlying mechanisms increased oxidative stress and endothelial dysfunction [102]. Besides many other proteins whose expression is influenced by high uric acid levels, enhanced alanine dehydrogenases (ALDR) was identified to mediate increased uric acid-induced ROS production in endothelial cells [59]. Komori et al. described that hyperuremic conditions increased ROS production in endothelial cells in vitro and thereby decreased PI3K/AKT signaling [61]. This subsequently reduced plasma membrane localization of breast cancer resistance protein (BCRP), a transporter protein that mediates the efflux of uric acid, thus further resulting in the intracellular accumulation of uric acid [61]. High intracellular levels of uric acid with subsequent ROS formation was also shown to increase ER stress and apoptosis indicated by increased levels of caspase-12 [60]. Decreased viability of endothelial cells in response to uric acid treatment was confirmed by Komori et al. [61]. Further, Li et al. reported that uric acid induced increased phosphorylation of eNOS at Thr^495^ mediated via PKC activation in response to ROS and ER stress. In addition, they showed a reduced binding of calmodulin (CaM) to eNOS upon uric acid treatment. Altogether, this resulted in a decreased eNOS activity and reduced NO production [60]. 

### 3.4. Discussion

CKD patients are at higher risk of developing CVD [104] and as kidney function declines, the risk of cardiovascular events increases and endothelial function decreases [50,105]. Given that endothelial dysfunction is key in the initiation and progression of atherosclerosis and contributes to decreased vascular reactivity, we investigated uremic toxin-induced endothelial dysfunction and its underlying mechanisms by means of a systematic review. Overall, cellular processes that were affected by uremic conditions or uremic toxins were inflammation, leukocyte migration and adhesion, oxidative stress, cell death, proliferation and thrombosis. Moreover, uremic toxins appeared to share common signaling pathways in endothelial cells, including pathways linked to MAPK, AhR, the RAGE receptor or pro-inflammatory transcription factors, for example NF-κB. As well as this, ROS as pro-inflammatory signal transducer was shared by multiple uremic toxins. 

p38-MAPK is a well-known mediator of pro-inflammatory cytokine expression and regulator of NF-κB activation. Uremic toxins such as IS signal via p38-MAPK/NF-κB to induce ICAM-1 or MCP-1 expression and thereby contribute to inflammatory responses of endothelial cells [38,42,44]. The role of NF-κB in uremic toxin-induced cytokine release was confirmed in macrophages by an IS-induced NF-κB and AhR dependent expression of TNF-α [40].

IS increases intracellular AhR expression in different cell types contributing to CVD progression [37,40,68,69]. Further, IS, as well as other uremic toxins derived from tryptophan metabolism, are ligands of AhR [106]. Activation of AhR signaling in endothelial cells was shown to enhance an inflammatory response as demonstrated by, e.g., increased levels of E-Selectin expression [37]. In CKD patients, serum E-Selectin was shown to be a predictor of cardiovascular events, with high levels of E-Selectin associated with a worse outcome [107]. Moreover, AhR as a contributor to CVD independent of CKD is described in several studies [108,109]. Furthermore, RAGE expression in endothelial cells was increased in response to uremic serum [30], AGEs such as CML [30,49,50] and pCS [30], inducing endothelial dysfunction and inflammation. In CKD patients, increased CML levels correlated with RAGE mRNA and VCAM-1 protein levels and inversely with endothelial reactivity [50]. In non-CKD patients, the association between increased RAGE expression and CVD is controversial [110], nonetheless, in vitro studies linked RAGE signaling to cardiovascular pathological processes also in the absence of CKD [111,112]. Combined, this suggests an amplification of both the AhR and RAGE pathways due to accumulation of uremic toxins in CKD and as such a larger contribution to inflammatory processes and the development of atherosclerosis.

Different uremic toxins such as IS, phosphate, cyanate, AGEs and uric acid led to a decreased eNOS expression and/or activity, resulting in a reduced production of NO and a decreased vasorelaxation [42,45,49,50,55,60]. With NO also being a thrombocyte inhibitor, decreased NO levels in combination with increased endothelial tissue factor and PAI expression in response to increased cyanate or carbamylated proteins such as cLDL, create a pro-thrombotic environment [45,47], which could contribute to the highly increased risk of thrombotic events in patients with CKD [113,114].

Furthermore, beyond the uremic toxins identified through our systematic literature search, the phosphaturic hormone fibroblast growth factor 23 (FGF23) is also highly increased in CKD and known to impair endothelial-dependent vasorelaxation. Underlying mechanisms include increased ROS production and reduced NO bioavailability, most likely independently of the FGF23 cofactor Klotho [115,116]. Instead, Klotho is reduced in CKD patients and mainly serves a protective role in endothelial cells by supporting NO production and vasorelaxation, as shown in both animal models of Klotho deficiency and overexpression as well as in vitro experiments [116,117,118,119,120]. Furthermore, Klotho is capable of reducing endothelial inflammation, also when induced by IS, [40,121,122] and has been shown to reduce endothelial permeability and apoptosis [123]. Notably, uremic toxins can reduce Klotho levels, as for example shown for AGEs in mouse podocytes [124] and for IS in SMCs [125].

A clear example of how post-translational modifications can contribute to atherosclerosis development and progression, is provided by the detrimental effects of oxidized LDL on the vascular wall, triggering endothelial dysfunction [12]. However, other post-translational modifications are also important in mediating CVD, with specific modifications catalyzed by uremic toxins [126]. Thus, the contribution of these post-translational modifications to CVD is expected to be of even greater relevance in CKD. In addition to affecting endothelial cell function, CKD-induced post-translational modifications also negatively affect other cell types important in CVD. For example, increased acetylation of LDL in CKD led to increased ER-stress induced apoptosis of macrophages [87]. Altogether, this shows that additional insights into CKD-induced post-translational modifications and their pathological effects regarding CVD are required to enable development of strategies to reduce CKD-induced post-translational modifications.

What is apparent from our literature search is that most studies investigated the effect of individual uremic toxins on single endothelial cellular functions or signaling pathways. Here, a proteomics, metabolomics or combined approach would provide a broad overview of cellular functioning and could aid in the discovery of novel mediators and signaling pathways involved. Further, while it is important to study the individual uremic toxin to elucidate the underlying signaling mechanisms, the vasculature of CKD patients is continuously exposed to a multitude of uremic toxins, which then combined contribute to endothelial dysfunction; thus, crosstalk between different toxins and their signaling pathways should also be investigated by combining toxins or using uremic serum or hemofiltrate obtained after dialysis of CKD patients, to study a broader spectrum of cellular effects induced by CKD-induced uremia. Of the identified studies, two studies investigated uremic serum as well as single toxins. Saum et al. showed that 10% uremic serum as well as CML-BSA equally decreased KLF2 expression; however, the effect of CML-BSA on ROS formation and monocyte adhesion was stronger than that of uremic serum [30]. This might be explained by the dilution of uremic serum in in vitro cell studies, resulting in lower concentrations of uremic toxins compared to patients with severe kidney failure. Furthermore, the composition of both serum and hemofiltrate differ drastically from plasma, due to coagulation in the case of serum preparation and, for hemofiltrate, due to the absence of larger uremic metabolites that are retained in the blood during dialysis as well as the low dialysis efficiency towards protein-bound uremic toxins. Overall, whether studying individual uremic toxins, toxin pools, uremic serum or hemofiltrate, there is no perfect solution to mimic CKD conditions in vitro; each approach has its benefits and drawbacks, and these should be considered based on the specific study aim when designing the experiment. 

This review underlines the importance of improving current dialysis treatments and developing novel, more efficient strategies to remove uremic toxins from the bloodstream and as such halt further uremic toxin-induced endothelial damage. Especially, protein-bound uremic toxins such as IS are notoriously difficult to dialyze and their accumulation has major consequences for endothelial health, as summarized in this review. As there is currently no universal technique that optimally removes all types of (protein-bound) uremic toxins, more research is needed to improve uremic toxin removal and thereby improve the cardiovascular health of CKD patients. In the past decade, there has been a focus on improved removal of protein-bound uremic toxins through adsorption techniques, as discussed in more detail elsewhere. This review highlights the signaling pathways frequently used by uremic toxins to exert detrimental cellular effects, as a potential complementary approach to reduce the cardiovascular burden of these uremic toxins in CKD.

## 4. Conclusions

In summary, this review connects current knowledge on pathophysiological and molecular mechanisms underlying increased cardiovascular risk in CKD, contributing to a deeper understanding of uremic toxin-induced signaling and providing indications on factors that should be considered in further analysis of uremic toxin-induced pathological effects. Overall, the accumulation of uremic toxins in CKD triggers endothelial dysfunction and contributes to inflammation, oxidative stress, thrombosis as well as cell death, thereby accelerating the development and progression of CVD. Uremic toxins frequently trigger ROS, MAPK/NF-κB, RAGE and/or AhR dependent pathways. Although these are already known in connection with the development of CVD also in the absence of CKD, this review clearly summarizes how uremic toxins accelerate or amplify these pathological mechanisms. Targeting these pathways, or interfering with uremic toxin accumulation or uremic toxins-induced post-translational modifications, may open therapeutic strategies to reduce the highly increased cardiovascular risk in CKD patients.

## Figures and Tables

**Table 1:** Effects of uremic toxins on key mechanisms in endothelial cells.

Toxin	Inflammation		Oxidative Stress		Cell Death		Migration/ Adhesion		Proliferation		Thrombosis	
Indoxyl sulfate	+	[37,38,39,40]	+	[31,37,38,39,41,42]	+	[31,40,42,43]	+	[37,39,40]	-	[31]		
Cyanate	+	[44,45]					+	[44]			+	[45]
cLDL	+	[46,47]			+	[47,48]	+	[46]				
AGE	+	[49]	+	[30,49,50]	+	[30]	+	[30,51]	-	[51]		
p-Cresol/ p-cresyl sulfate	+	[52]	+	[30,31,52,53]	+	[30,31,54]	+	[30,52]	-	[31]		
Phosphate			+	[55]	+	[55,56]					+	[57]
ADMA					+	[58]						
Uric acid			+	[59,60,61]	+	[60,61]						

(+) increased; (-) decreased effect. ADMA = asymmetric dimethylarginine; AGE = advanced glycation end products; cLDL = carbamylated LDL; LDL = low density lipoproteins.

## Supplementary Materials

The following are available online at https://www.mdpi.com/article/10.3390/ijms23010531/s1.
